# Air Pollution Exposure and the Relative Risk of Sudden Sensorineural Hearing Loss in Taipei

**DOI:** 10.3390/ijerph19106144

**Published:** 2022-05-18

**Authors:** Chun-Gu Cheng, Yu-Hsuan Chen, Shang-Yih Yen, Hui-Chen Lin, Hung-Che Lin, Kuei-Ru Chou, Chun-An Cheng

**Affiliations:** 1 Department of Emergency, Taoyuan Armed Forces General Hospital, Taoyuan 32549, Taiwan; doc50015@yahoo.com.tw; 2 Department of Emergency Medicine, Tri-Service General Hospital, National Defense Medical Center, Taipei 11490, Taiwan; 3 Emergency Department, Department of Emergency and Critical Medicine, Wan Fang Hospital, Taipei Medical University, Taipei 11696, Taiwan; 4 Department of Emergency, School of Medicine, College of Medicine, Taipei Medical University, Taipei 11031, Taiwan; 5 Division of Chest Medicine, Department of Internal Medicine, Cheng Hsin General Hospital, Taipei 11220, Taiwan; anemia0829@gmail.com; 6 Department of Neurology, Tri-Service General Hospital, National Defense Medical Center, Taipei 11490, Taiwan; shangyih@yahoo.com.tw; 7 School of Nursing, College of Nursing, Taipei Medical University, Taipei 11031, Taiwan; ceciliatsgh@gmail.com (H.-C.L.); kueiru@tmu.edu.tw (K.-R.C.); 8 Department of Otolaryngology-Head and Neck Surgery, Tri-Service General Hospital, National Defense Medical Center, Taipei 11490, Taiwan; lhj50702@gmail.com; 9 Center for Nursing and Healthcare Research in Clinical Practice Application, Wan Fang Hospital, Taipei Medical University, Taipei 116, Taiwan; 10 Department of Nursing, Taipei Medical University-Shuang Ho Hospital, New Taipei 23561, Taiwan; 11 Psychiatric Research Center, Taipei Medical University Hospital, Taipei 110301, Taiwan; 12 Neuroscience Research Center, Taipei Medical University, Taipei 11031, Taiwan

**Keywords:** air pollution, sudden sensorineural hearing loss, lag effect

## Abstract

(1) Background: The etiologies of sudden sensorineural hearing loss (SSHL) remain unclear. The level of mean particulate matter with a diameter of 2.5 μm or less (PM2.5) was not associated with SSHL, but the maximum PM2.5 level exhibited a negative association with SSHL in Korea. Exposure to nitrogen dioxide (NO_2_) for 2 weeks increased the risk of SSHL. The lag effects of SSHL after air pollution exposure were limited. We aimed to evaluate the association of SSHL with air pollution exposure to determine whether air pollution exposure caused delayed effects. (2) Methods: This observational study used inpatient data obtained from electronic health records at the Tri-Service General Hospital from 2011 to 2019. The data of all SSHL patients were retrieved. The air quality dataset from Songshan station from 2011 to 2019 was used. The main outcomes were the relative risks (RRs) of SSHL associated with PM2.5, O_3_, and NO_2_ exposures within 1 month. The relationships between these factors were examined using distributed lag nonlinear time series models. (3) Results: The RR of SSHL associated with PM2.5 exposure was 1.195 (95% confidence interval (C.I.: 1.047–1.363) for a 10 unit increase at a lag of 7 days. The RR of SSHL associated with O_3_ exposure was 1.14 (95% C.I.: 1.003–1.3) for a 10 unit increase at a lag of 9 days. The RR of SSHL associated with NO_2_ exposure was 1.284 (95% C.I.: 1.05–1.57) for a 10 unit increase at a lag of 23 days. (4) Conclusions: In our study, SSHL was confirmed to be associated with air pollution exposure with a lag effect. We discussed possible mechanisms to explore possible biological hypotheses and support further research. Large-scale studies including participants with other ethnicities and causal relationships are needed to confirm our findings.

## 1. Introduction

Sudden sensorineural hearing loss (SSHL) is characterized by sudden-onset hearing loss, and in approximately 90% of patients, significant idiopathic causes cannot be identified. SSHL affects 5 to 27 per 100,000 people annually [1,2,3], with approximately 66,000 new cases per year in the United States [4]. SSHL is a type of hearing loss that (a) is sensorineural in nature, (b) occurs within a 72-h window, and (c) meets certain audiometric criteria. The most frequently used audiometric criterion for SSHL is a more than or equal to 30-decibel decrease in hearing that affects at least 3 consecutive frequencies [5].

Although viral infection, environmental factors, occupational factors (such as exposure to loud noise, heavy metals, and organic solvents), autoimmune diseases, cardiovascular diseases, accidental events, endothelial dysfunction, metabolic diseases, and health habits (such as smoking and alcohol consumption) are risk factors for SSHL, the complex etiologies of SSHL remain unclear [6].

Many studies have found that air pollution is related to cardiovascular diseases, respiratory diseases, and mortality [7,8,9]. A previous study found that the maximum mean particulate matter with a diameter of 2.5 μm or less (PM2.5) was negatively associated with SSHL in Korea [10]. Individuals with long-term moderate or high levels of exposure to PM2.5 had a higher risk of SSHL development than individuals with a low level of exposure in Taiwan [11]. The group with a fourteen-day exposure to NO_2_ had an increased risk of SSHL compared with the control group in Korea [12]. Individuals with long-term higher levels of exposure to NO_2_, nitrogen oxide, and CO carry a higher risk of developing SSHL in Taiwan [11]. There is a delayed effect associated with air pollution exposure and disease development. Information on the lag effects of air pollution on SSHL is worthy of study using time series methods.

We hypothesized that SSHL was related to air pollution in Taipei. We retrieved daily SSHL hospitalization data from electronic medical records at the Tri-Service General Hospital from 2011 to 2019. A distributed lag nonlinear model was used to evaluate the relative risk (RR) of SSHL hospitalization associated with exposure to air pollution. We reviewed the possible pathway of air pollution causing SSHL to explore possible biological hypotheses. It is important for healthcare providers to understand the lag effects of exposure to air pollution and for the government to promote aggressive air pollution control policies to reduce the risk of SSHL. The possible mechanisms of the relationship between air pollution and SSHL must be confirmed in the future according to the findings in this paper.

## 2. Materials and Methods

**Figure 1:**
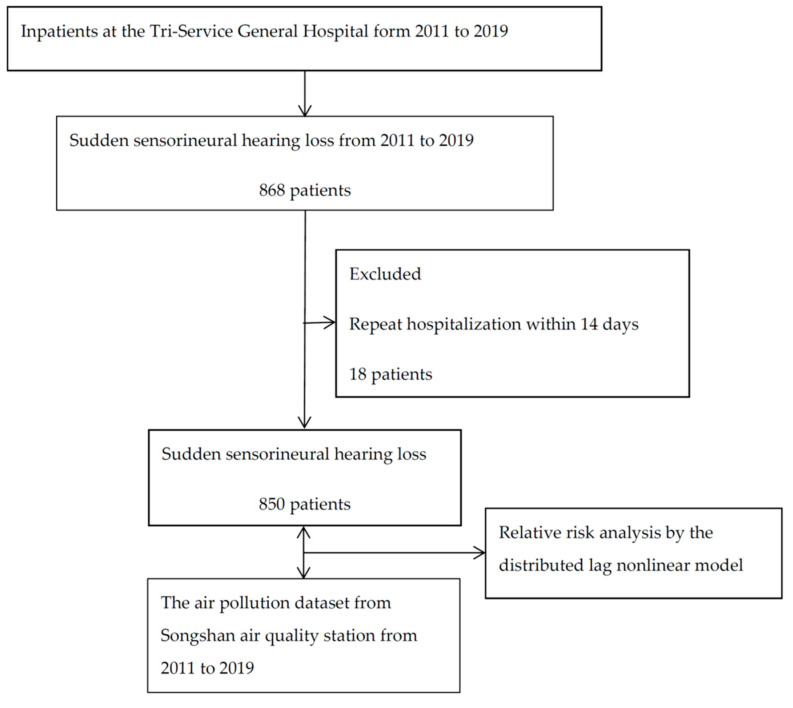
The flowchart for this study.

We analyzed daily SSHL admission data from the Tri-Service General Hospital in Taipei, Taiwan, and an inpatient dataset from 2011 to 2019. SSHL was confirmed by audiometry and defined as a 30 dB loss over three contiguous frequencies occurring within 3 days before visiting our hospital and no mass or infarcted area detected on magnetic resonance imaging. The hospital is in the eastern region of Taipei city. A total of 61,554 patients were discharged or died in 2019. The NeiHu District is located in the eastern region of Taipei and had 281,615 residents in 2021. The International Classification of Diseases, 9th Revision, Clinical Modification (ICD-9-CM) code 388.2 and ICD-10-CM code H91.20 were used to identify SSHL patients. We excluded patients with repeat hospitalizations within 2 weeks for the same disease and those with a history of administration of ototoxic drugs for infectious, traumatic, vascular or hematologic, and neoplastic disorders. The flowchart is shown in Figure 1. The study was approved under approval number TSGHIRB No.: C202105149.

The concentrations of PM2.5, NO_2_, O_3_, sulfur dioxide (SO_2_), and CO as well as ambient temperature and relative humidity data were obtained from the Taiwanese Environmental Protection Administration (Table 1) [13]. The air quality monitoring station in Songshan is located at 25°04′50″ N, 121°58′05″ E, and the distance between the monitoring station and the Tri-Service General Hospital is approximately 2.81 km according to Google Maps.

Descriptive statistics of air pollutants and meteorological factors were analyzed. Poisson correlation was performed to evaluate the relationships between air pollution and meteorological factors. To reduce the likelihood of a multiple collinearity problem, we screened variables by setting the inclusion criteria at |r| < 0.8.

To study the association between SSHL hospitalization and air pollutants (PM2.5, PM10, O_3_, CO, NO_2_, and SO_2_), we performed a distributed-lag model analysis (DLMN) [12]. The DLMN described associations with potentially nonlinear and delayed effects in the time series data. The RR for admission due to SSHL associated with the level of ambient pollution was determined using a quasi-Poisson regression model. Quasi-Poisson regression was used to evaluate the number of cases associated with other variables (air pollutants, days of the week, air temperature), not the causal relationship. Poisson regression is a generalized linear model for count data and assumes that the response variable Y and the logarithm of its expected value can be modeled by a linear combination of air pollutants parameters. We used a linear function for air pollutants in the exposure–response function. We applied a maximum lag of up to 30 days for each examined pollutant to estimate the RR. In the DLMN, a natural cubic spline with four equally spaced internal knots was used for temperature and relative humidity. A natural cubic spline for the calendar time with 7 degrees of freedom (df) was applied for every year to adjust for seasonality and time trends. We also adjusted the factor variables for days of the week. Threshold values were set as the mean of each air pollution variable. RRs are shown as a 10 unit increase from the mean of each air pollutant (reference) during a statistically significant lag time. The R package dlnm (R version 4.0.2.) was used for analysis [14].

## 3. Results

In total, 850 SSHL patients were admitted from 1 January 2011 to 31 December 2019. There were 466 male and 384 female patients, and the mean age was 49.69 ± 16.6 (range 6–90) years. There were 109 SSHL patients with hypertension, 96 patients with diabetes mellitus, 30 patients with hyperlipidemia, and 21 patients with chronic kidney disease. The hospitalization rate and prevalence of SSHL in our study were 138/100,000 and 30/100,000, respectively.

**Figure 2:**
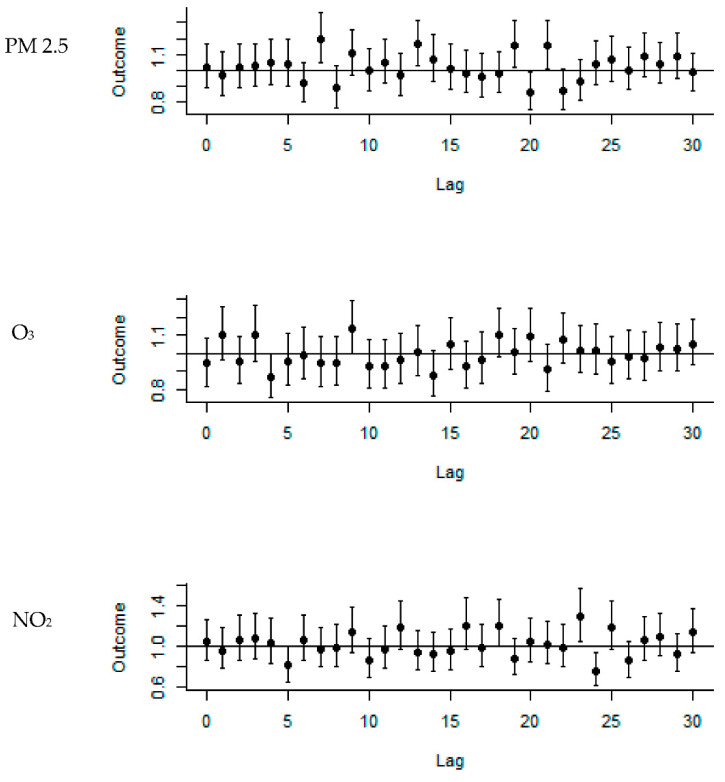
The lag effects of SSHL after air pollution exposure.

The collinearity tests showed results greater than 0.8 for PM2.5 and PM10, as well as for NO_2_ and CO, so we excluded PM10 and CO from the analysis (Table 2). The RR of SSHL associated with PM2.5 exposure was 1.195 (95% confidence interval (C.I.: 1.047–1.363) for a 10 unit increase at a lag time of 7 days. The RR of SSHL associated with O_3_ exposure was 1.14 (95% C.I.: 1.003–1.3) for a 10 unit increase at a lag of 9 days. The RR of SSHL associated with NO_2_ exposure was 1.284 (95% C.I.: 1.05–1.57) for a 10 unit increase at a lag of 23 days (Figure 2).

## 4. Discussion

Our study revealed that the lag effects of exposure to air pollution (PM2.5, O_3_, NO_2_) were associated with daily numbers of hospitalizations due to SSHL in Taipei. SSHL affects the daily life of patients and results in substantial health expenses. Air pollution is a global issue that is worsened due to industrial processes in developing countries. The positive relationship between SSHL and air pollution needs to be considered in the prevention of SSHL.

Taipei is located in a basin, causing air pollution to settle because it is not removed by wind, resulting in prolonged exposure for residents. Taipei is the capital of Taiwan, with 2,602,418 residents, 815,343 cars (313 cars for every thousand persons), and 946,851 motorcycles (364 motorcycles for every thousand persons), causing heavy traffic in 2020 [15]. Hyperbaric oxygen therapy is performed in our hospital, and the number of patient hospitalizations for oxygenation therapy is high.

SSHL is related to multiple factors, such as infection, metabolic causes, vascular impairment, inner ear pathologies, and autoimmune diseases [2,3]. Air pollution induces SSHL through multifactorial mechanisms, including toxicity, oxidative stress, and the upregulation of inflammatory pathways. The possible mechanisms of hearing damage after air pollutant exposure are as follows: inhaled air pollutants cause the disruption of the redox signal, oxidative stress with an oxidant and antioxidant imbalance, and neuroinflammation [16]. The blood–brain barrier (BBB) separates blood and extracellular fluid and protects the brain from any external toxins. Reactive oxygen species (ROS) lead to overproduction-induced mitochondrial DNA deletion, causing dysfunction, inhibiting creatine kinase activity and P53 expression, causing deficiencies in energy transfer, and inducing apoptosis by inhibiting the bcl-2 protein in the mitochondrial membrane [17]. ROS also induce blood flow insufficiency in the cochlea. Endothelial dysfunction decreases perfusion by decreasing nitric oxide and increasing interleukin expression and nuclear factor activities. Previous studies have confirmed that oxidative stress induced SSHL by stimulating endothelial dysfunction of the inner ear microcirculation [18].

The oxidative stress increased by the generation of ROS and reactive nitrogen species induces chronic inflammation, lipid denaturation, microglial dysfunction, and white matter demyelination, leading to the progression of neurological disorders. Brain tissue that was exposed to air pollution had elevated IL-1β expression, had an increased number of infiltrating monocytes in the microglia, had a disrupted BBB, and showed influenced endothelial cells [19]. Tumor necrosis factor-alpha (TNF-α) is an important pathological change in cochlear microcirculation that alters the sphingosine-1-phosphate signaling pathway [20]. A previous study found higher neutrophil and lymphocyte ratios in the SSHL group compared with normal group, indicating an increased inflammatory state [21]. Inflammation in the respiratory tract after air pollution exposure increased proinflammatory cytokines; systemic inflammation then affected components of the central nervous system, such as IL-1 [18] and TNF-α [19], and then upregulated COX-2 [22] and induced neuroinflammation of the capillary endothelium in the brain.

The gases and black carbon were composed of PM2.5 fractions. Penetrating PM enters lung cells, macrophages, and the blood. PM mainly influences the respiratory system with the injury of lung tissue cells and downregulates immune defense mechanisms in the lungs [23]. PM2.5 exposure increased the risks of respiratory syncytial virus and influenza virus infections in Italy [24] and China [25]. PM absorption initially disrupts the BBB through a cluster of differentiation, signaling, and inflammatory activities that directly influence the brain [26]. A previous study found no association between SSHL and the mean PM2.5 value and explored a negative association between SSHL and the maximal PM2.5 value in Korea [9]. Our study found that PM2.5 had a lag effect on SSHL and extended the PM2.5 delay effect on SSHL. Potential reasons include heavy traffic and difficulty in removing PM2.5 via wind due to the terrain of Taipei, with persistent toxic effects rather than higher wind effects in Korea.

SSHL was associated with a lag effect of the 14 day NO_2_ concentration (0.1 ppm) with an odds ratio (OR) of 3.12 in Korea [12]. Our study also discovered a similar finding, with a lag effect of 23 days, indicating a chronic effect. A previous study found that long-term exposure to NO_2_ increased the rate of acute upper respiratory infections (URIs), with an RR of 1.25 associated with 6 days of cumulative exposure; there was also a prolonged lag effect for SSHL in Taiwanese children who were easily injured [27] and a lag effect associated with 2 to 6 weeks of exposure in adults according to an international atherosclerosis survey [28]. URI was a risk factor for SSHL, and the delayed effect of NO_2_ may be mediated in this way.

O_3_ is a double-sided blade as it can kill viruses, but it increases inflammation in the lungs, resulting in harmful lung disease. A recent study found that animals exposed to O_3_ and PM had increased nuclear factor-κB activation of astrocytes, glial cells, and endothelial cells expressed by inducible nitric oxide synthase, an increase in ROS production, and protein expression changes by BBB disruption in cerebral capillaries. The peripheral cytokines increased by air pollutants caused BBB dysfunction with damage to endothelial cells, glial cells, and neurons through the enhancement of the expression of cytokine receptors in the endothelium of cerebral capillaries [29]. PM2.5 had higher RRs in lags of 13, 19, and 21 days, but O_3_ did not show a greater lag effect due to a balanced risk of anti-inflammation and eliminating viruses.

URIs related to PM2.5, NO_2_, and O_3_ exposure generally lasted for 6 days in children in Taipei [27]. The risk of URI in adults increased after 2–6 weeks of air pollution exposure [28]. The level of cytokines persisted for approximately 4 days after influenza infection in an animal study, and inflammation and oxidative stress were elevated for several days and eventually influenced hearing [30]. Hypoperfusion of the cochlea was subacute, and chronic immune-mediated inflammation was observed. Our study found that all three pollutants had delayed effects of SSHL at more than one week; a potential reason for this was another main effect of air pollution associated with URIs that affected SSHL. Further study is needed to confirm this hypothesis.

In patients with severe infection, such as sepsis, inflammation can cause sensorineural hearing loss [31] and ischemic stroke [32]. SSHL is an immune-mediated disease that responds to steroids, but it is not present in all cases of autoimmune disease. SSHL is associated with viral infection and is thought to be induced by viral toxic reactions, immune-mediated responses, or stress responses that induce inflammation [33]; however, only some SSHL patients reported previous recent viral infections (4.1% in China) [34]. There is no association between SSHL and viral infection due to widespread vaccination policies worldwide. Taiwan has 95% vaccination coverage in children (before starting school), approximately 80% coverage in students (before starting university), and 50% coverage in older people. The influenza vaccine, with which 28% of all citizens in Taiwan have been vaccinated [35], with the majority of vaccinated individuals being young, older and at higher risk, was found to reduce influenza infection. Influenza vaccination reduced outpatient visits for URIs, cardiovascular disease incidents, and ischemic stroke, as proven in previous studies [36,37]. The SSHL group had a mean age of 50 years, was mostly nonvaccinated for influenza, and had possibly a higher rate of influenza-infection-mediated SSHL in our study.

Neuroinflammation and oxidative stress can act upon the apoptotic pathway and induce neuronal loss. A recent study suggested that oxidative stress and inflammatory responses after inhaling air pollutants increased the risk for cerebral ischemia by stimulating reactive protein expression with infiltration with leukocytes and inflammatory cell adherence-obstructing vessels. The diseases caused defects of the vascular system in the cochlea by microcirculation ischemia. The mechanism of vascular problems includes atherosclerosis, hypotension, and vasospasm [38]. A recent study found that SSHL was related to cardiovascular factors and that the risk of cardiovascular diseases increased after air pollution exposure [39]. The delayed effects of air pollution exposure are worthy of study. NO_2_ is an indicator of traffic pollution in urban regions. The effect of NO_2_ on acute myocardial infarction was greater than that of PM10 and O_3_ in a past study [40].

A past study found adverse effects of CO and NO_2_ in participants with long-term sensorineural hearing loss. High-level CO exposure compared with low-level CO exposure increased the risk of sensorineural hearing loss. Compared with low-level NO_2_ exposure, mid-level and high-level NO_2_ exposures increased the risk of sensorineural hearing loss [41]. However, our study found a collinear effect of NO_2_ and CO, and we excluded CO from our study.

**Figure 3:**
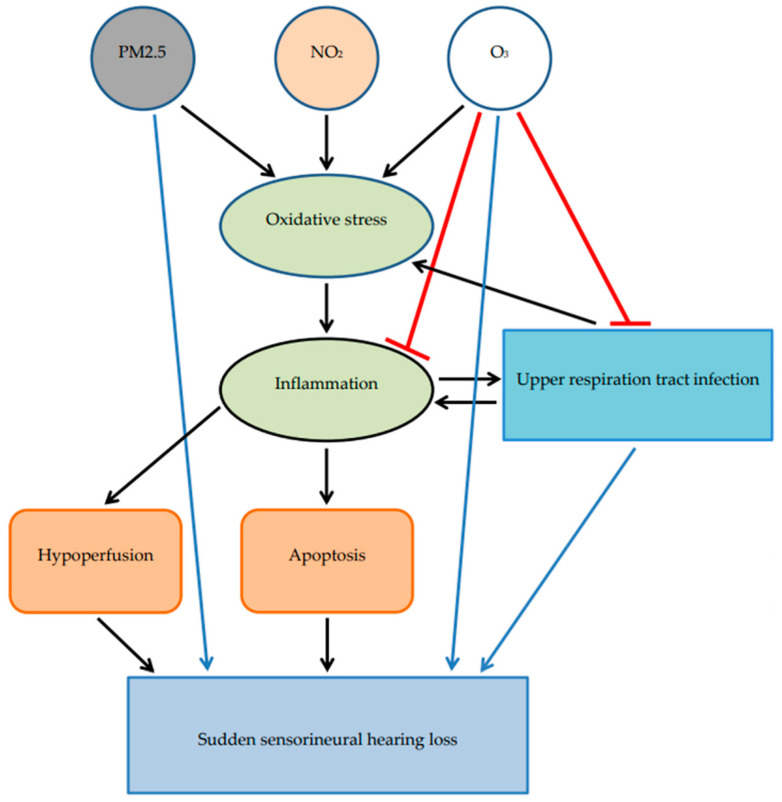
Possible mechanisms associated with air pollutants. The neurotoxicity of PM2.5 and O_3_ directly affects cochlear cells (blue line). PM2.5, NO_2_, O_3_ and upper respiration tract infection induce oxidative stress in the inflammatory pathway and cause apoptosis and hypoperfusion. Inflammation and upper respiration infection could interact and cause each other occur. (black line). PM2.5, NO_2_, and O_3_ decrease the defenses of the upper respiratory tract, facilitating viral infections. Viruses directly infect cochlear cells (blue line) and induce oxidative stress and inflammation, causing SSHL. O_3_ has an anti-inflammatory effect and can kill viruses (red line) via the confrontation effect [28,29,30,31].

Our results support the assumption of an association between SSHL and several weeks of air pollution exposure according to data from a single medical center. Physicians and patients need to increase their awareness of this possible disease that is induced by air pollution. The possible mechanisms associated with the three air pollutants in this study are summarized in Figure 3. The rapid diagnosis of SSHL is imperative to initiate appropriate treatment, and effective strategies to reduce air pollution should be developed by the air quality department of the government. Sudden deafness is an emergency otolaryngological condition. It is best to start active treatment within 7–14 days to help the patient achieve the greatest hearing recovery possible.

There are some limitations in this study. First, atherosclerosis [42], migraine [43], and thyroid disease [44] related to SSHL were not evaluated. The comorbid conditions of SSHL patients were not included in this study as adjustment factors when assessing the relationship with SSHL by quasi-Poisson regression due to the main target of this study being the lag effect of air pollution exposure related to SSHL. The causal relationship using a control group must be analyzed in future studies. Second, the majority of SO_2_ concentrations were below 10 ppb, and we did not evaluate their effects on SSHL. We analyzed the effect of SO_2_ concentrations in 10 unit increments; however, increases of more than 13 ppb were rare. Third, there was a small proportion of SSHL patients with evidence of ischemic stroke (6/850), and the relationship with the vascular pathway needs further study.

## 5. Conclusions

This is the first study to confirm the delayed effects of air pollution exposure related to SSHL. Despite the annual reduction in air pollution in Taipei due to the cooperation of the government and residents, the relatively high levels of PM2.5, O_3_, and NO_2_ were still harmful and associated with SSHL incidents. These results broaden the knowledge about the lag effects of air pollution associated with the incidence of SSHL. Public education about the risk of SSHL should be provided and mask use encouraged, and more effective policies to decrease air pollution should be implemented by the government. Larger studies with survey population data are needed to confirm this finding.

## Figures and Tables

**Table 1:** Air pollutants and meteorological factors recorded at the Songshan air quality station over 9 years (2011–2019).

Pollutant	Mean ± Standard Deviation	25%	50%	75%	Minimum	Maximum
PM2.5 (ppm)	18 ± 10.64	12	18	26	2	85
PM10 (ppm)	36.57 ± 18.15	24	36.57	45	5	147
O_3_ (ppm)	25.6 ± 10.39	19.2	25.6	32.8	2.8	90
NO_2_ (ppb)	19.83 ± 7.26	15.86	19.83	24.26	2.38	65.63
SO_2_ (ppb)	2.6 ± 1.54	1.9	2.6	3.6	0.2	20.8
CO (ppm)	0.46 ± 0.2	0.37	0.46	0.58	0.08	2.63
Air temperature (°C)	23.79 ± 5.55	19.03	23.79	28.48	5.5	33.3
Relative Humidity (%)	73.42 ± 9.54	66.34	73.42	81	24.57	95.01

PM2.5: mean particulate matter with a diameter of 2.5 μm or less; PM10: mean particulate matter with a diameter of 10 μm or less; O_3_: ozone; NO_2_: nitrogen dioxide; SO_2_: sulfate dioxide; CO: carbon monoxide; ppm: parts per million; ppb: parts per billion.

**Table 2:** Correlation analysis of all air pollutants and meteorological factors.

	PM2.5	PM10	O_3_	NO_2_	SO_2_	CO	Temperature	RH
PM2.5		0.843 ***	0.233 ***	0.520 ***	0.441 ***	0.557 ***	−0.127 ***	−0.222 ***
PM10	0.843 ***		0.348 ***	0.451 ***	0.384 ***	0.473 ***	−0.186 ***	−0.359 ***
O_3_	0.233 ***	0.348 ***		−0.126 ***	0.001	−0.187 ***	−0.173 ***	−0.266 ***
NO_2_	0.52 ***	0.451 ***	−0.126 **		0.456 ***	0.859 ***	−0.273 ***	0.117 ***
SO_2_	0.441 ***	0.384 ***	0.001	0.456 ***		0.333 ***	0.174 ***	−0.157 ***
CO	0.557 ***	0.473 ***	−0.187 ***	0.859 ***	0.333 ***		−0.167 ***	0.103 ***
Temperature	−0.127 ***	−0.186 ***	−0.173 ***	−0.273 ***	0.174 ***	−0.167 ***		−0.254 ***
Relative Humidity	−0.222 ***	−0.359 ***	−0.266 ***	0.117 ***	−0.157 ***	0.103 ***	−0.254 ***	

Note: *** *p* < 0.001; ** *p* < 0.01. PM2.5: mean particulate matter with a diameter of 2.5 μm or less; PM10: mean particulate matter with a diameter of 10 μm or less; O_3_: ozone; NO_2_: nitrogen dioxide; SO_2_: sulfate dioxide; CO: carbon monoxide.

## Data Availability

The datasets used in the current study are available from the corresponding author upon reasonable request.
